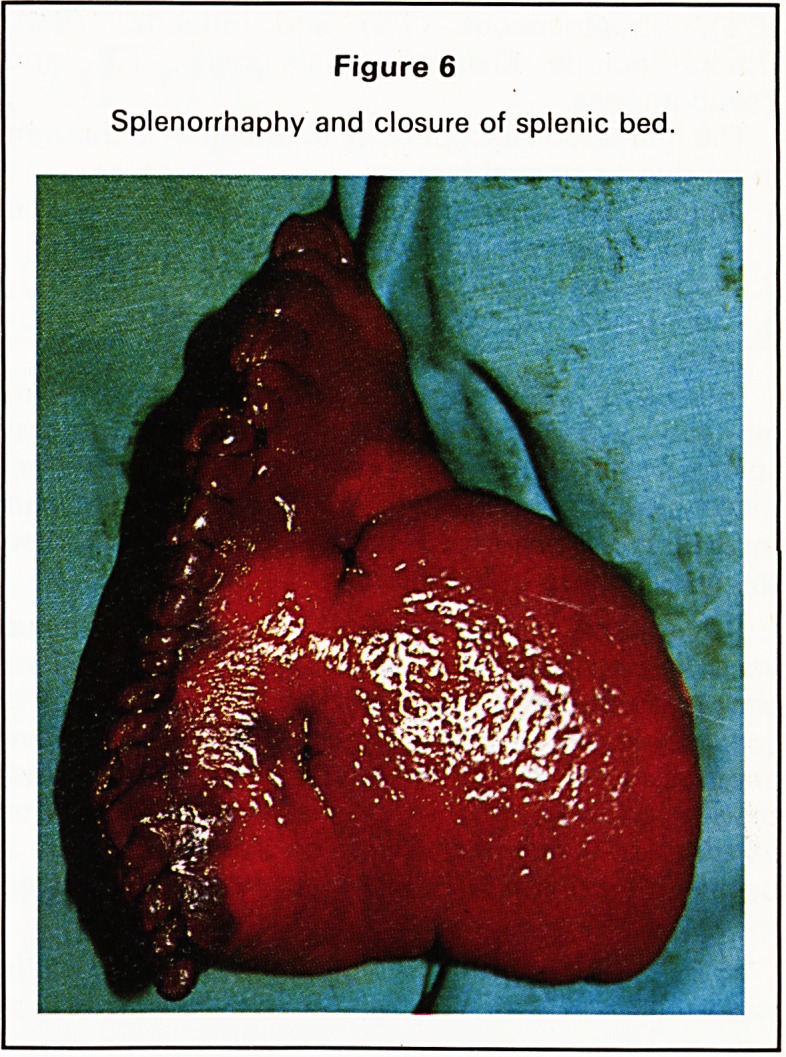# Splenic Cyst, the Case against Splenectomy

**Published:** 1984-10

**Authors:** Ahmed A. Shandall, Jonathan M. Frappel, Roger L. Celestin

**Affiliations:** Department of Surgery, Frenchay Hospital, Bristol; Department of Surgery, Frenchay Hospital, Bristol; Department of Surgery, Frenchay Hospital, Bristol

## Abstract

Splenectomy is the established treatment for symptomatic splenic cysts. The high incidence of overwhelming post-splenectomy infection calls for a review of this treatment. A case of post-traumatic pseudocyst treated by splenic cystectomy, without sequelae, is reported. A plea is made for salvaging the spleen, whenever possible.


### Splenic Cyst: The Case against Splenectomy

**Ahmed A. Shandall, F.R.C.S.**
Registrar
**Jonathan M. Frappel, F.R.C.S.**
Senior House Officer
**Roger L. Celestin, F.R.C.S.**
Consultant
Department of Surgery, Frenchay Hospital, Bristol

'Este Igitur Splenatum Necessarius?' Guliemi Ballonii, 1578.

### SUMMARY

Splenectomy is the established treatment for symptomatic splenic cysts. The high incidence of overwhelming post-splenectomy infection calls for a review of this treatment. A case of post-traumatic pseudocyst treated by splenic cystectomy, without sequelae, is reported. A plea is made for salvaging the spleen, whenever possible.

### INTRODUCTION

Zaccarelli in 1549 reported on splenectomy, but on the same subject in 1578 Ballonii asked the question 'Is the spleen so necessary for life?'; while the first successful partial splenectomy was carried out by Timothy Clark in 1673, and the first total one in 1678 by Nicholaus Matthias.

Splenectomy as the recommended treatment for splenic injury or lesions is firmly rooted in surgical teaching being held to be without harmful sequelae.

Kocher in 1911 said: 'Injuries of the spleen demand excision of the gland. No evil follows its removal, while danger of haemorrhage is effectively stopped.'[1]

Hamilton Bailey takes up the theme in 1927 adding 'in no instance is there the slightest indication that a splenectomised person is more susceptible to infection than the rest of humanity'.[1]

McIndoe in 1932 rejects efforts at preserving the spleen: 'the operation for rupture of the spleen is splenectomy. Any form of repair or tamponade is inadvisable.'[1]

Yet as early as 1919 Morris and Bullock[2] had showed an increase in infection in splenectomised rats exposed to the rat plague bacillus. This evidence remained ignored until King and Schumacker[3] in 1952 presented five infants who developed severe sepsis after splenectomy for hereditary spherocytosis.

Today the major role played by the spleen in host defence to infection is no longer controversial and has been fully reviewed by Singer in 1973[4] who showed that the mortality rate from sepsis following splenectomy is 200 times that of the general population.

With such evidence preservation of the spleen is not only an ethical issue but is likely to become an important medico-legal one that can no longer be ignored.

### CASE REPORT

Mrs. S. T., aged 36, presented in September 1981 with epigastric and left chest pain. Physical examination revealed a palpable spleen. In 1969 she had a road traffic accident and was unconscious for 2 days. In 1978 she had a further RTA with a 'steering wheel injury'. In 1980 she had fallen heavily downstairs.

A barium meal showed displacement of the stomach to the right. An ultrasound examination suggested a large splenic cyst (Figure 1); this was confirmed on arteriography (Figure 2). Laparotomy was performed in October 1981 via a left paramedian incision and revealed a large cyst of the upper pole of the spleen attached to the under surface of the diaphragm. A table cystogram (Figure 3) was performed to define the confines of the cyst which was then emptied. It contained 2 litres of clear fluid. The spleen was then totally mobilised and carefully exteriorised on its vascular pedicle. Enucleation of the cyst was then undertaken and it was finally excised (Figure 4) leaving a bed in which small vessels were easily ligated (Figure 5). Bleeding was not a problem. The bed of the cyst was then folded

**Figure 1**

Ultrasound suggesting a large cyst at the upper pole of the spleen.

**Figure 2**

Arteriogram showing stretching of the branches of the superior division of the splenic artery.

**Figure 3**

Table cystogram defining the large size of the cyst.

upon itself and the spleen was reconstituted with horizontal mattress catgut sutures (Figure 6) so as to obliterate the space within the fold. The spleen was then returned to its natural site without drainage.

She made an uninterrupted recovery and 16 months after her operation remains well and asymptomatic. Scanning of the spleen 6 months post-operatively shows the spleen to be of normal contours with no change in its size or shape.

### DISCUSSION

This case is presented as a plea to avoid splenectomy with its consequent complications – sometimes fatal; and to suggest splenic cystectomy or splenorrhaphy whenever possible; or when not possible to discuss additional measures that should, of necessity, follow splenectomy.

Péan, in 1867, was the first to attempt the resection of a splenic cyst, but was unsuccessful. Since, cysts have been treated by splenectomy.[5]

**Figure 4**

Walls of enucleated cyst.

**Figure 6**

Splenorrhaphy and closure of splenic bed.

**Figure 5**

Bed of cyst, showing a 'dry' field.

Recent reports have appeared suggesting treatment by partial splenectomy,[6] or by partial excision and drainage.[7] No report of excision and splenorrhaphy has been reported to date.

Singer in 1973[4] defined overwhelming post-splenectomy infection as 'septicaemia, meningitis, or pneumonia usually fulminant'. In his review of 2795 cases he showed a sepsis rate of 4.25% with a mortality of 2.52%, that is 200 times that of the general population. This occurred in both adults and children regardless of whether there was underlying disease or not. The mortality from sepsis was 0.58% in post-traumatic cases (×58 G.P.) and 0.86% in incidental splenectomy (×86 G.P.). Half of the fatal cases were encountered in the first 12 months, with most within 3 years, the longest time lag being 25 years. Fifty-eight per cent of all septic cases were fatal, the cause of death being usually disseminated intravascular coagulation and acute bilateral suprarenal haemorrhage (Waterhouse–Friderichsen syndrome).

In another follow-up over a 30-year period involving 740 war veterans who had lost their spleens, Robinette[8] showed a high mortality rate from pneumonia. A wide spectrum of organisms is responsible for sepsis,[4] with Pneumococcus (48%) and Meningococcus (12%) accounting for the majority; and less frequently *E. coli* (11%), Staphylococcus

(8%), Streptococcus (7%) and influenza (8%). Others include Klebsiella, Salmonella, T.B. and Pseudomonas.

The increased susceptibility to infection is thought to be due to several factors:

1. Impairment of initial response to bacteria and other particulate antigens in the blood stream.[9]
2. Depression of IgM level.[10]
3. Deficiency of phagocytosis-promoting peptides.[11]

In view of such morbidity and its high attendant mortality, every effort should now be made to resist splenectomy whenever possible. Either splenorrhaphy or partial splenectomy being carried out instead. If splenectomy is inevitable in an adult, then the following precautions must be taken:

(a) *Elective splenectomy*. Give Pneumococcus vaccine *preoperatively* as response to antigens should be good and may last up to 8 years. This is specially important if the patient is intended to have chemotherapy after splenectomy when the response then is very poor. In addition, antibody level prior to vaccination is worth determining as a low level is a useful indicator to the need for yet more prophylactic therapy.

(b) *Essential splenectomy*. Give Pneumococcus vaccine (Pneumovax) up to three doses with great care; and Penicillin V, 250 mg four times a day for 3 months. It has been argued that antibiotic therapy should be kept up for 3 years, but compliance becomes a problem and the advantages of this longer term are still controversial.[4,12] In penicillin-allergic patients, trimethoprim/sulfamethoxazole (Septrin) is substituted.

Failure to take such precautions could easily be argued as negligence and become a serious medico-legal problem to the unwary.

The protagonists of splenectomy base their reluctance to salvaging the spleen on the hypothetical complications of delayed bleeding with delayed rupture or splenic pseudocyst formation. However, there has been no report of these following splenorrhaphy. Others argue that bleeding cannot always be controlled during repair, which is a tedious procedure. In fact, bleeding can be controlled by temporary clamping of the splenic artery or, if necessary, ligation of that artery or its branches[13]; but it demands that the operator be familiar with its anatomy. Michels' description[14] of the anatomy of the spleen (1942) deserves studying, though it suffices to remember simply that the arterial pressure within the viscus depends virtually on the splenic artery which bifurcates OUTSIDE the spleen thus allowing control of a particular segment. These two arteries then branch longitudinally, with transverse septa then crossing the spleen. Lacerations usually follow the arterial pattern. Such a simple anatomical framework should not present difficulties to a competent surgeon – and repairs of the spleen are not new. The first one was reported in 1902 by Berger followed by another in 1910 by Mayo.[15] More recently Morgenstern (1966)[16] and Mishalany[17] (1974) have described partial splenectomy and repairs, and several more reports have followed since.[18,13]

Though controlled studies of the overwhelming post-splenectomy infection syndrome (OPSI) have yet to be undertaken, we believe that a new era in surgery of the spleen has begun and that its preservation should now form part of good surgical practice.

### REFERENCES

1. SHERMAN, R. (1980) Perspectives in management of trauma to the spleen. *The Journal of Trauma* **20**, 1–13.
2. MORRIS, D. H. and BULLOCK, F. D. (1919) The importance of the spleen in resistance to infection. *Ann.Surg.* **70**, 513–521.
3. KING, H. and SCHUMACKER, H. B. JR. (1952) Splenic studies: I. Susceptibility to infection after splenectomy performed in infancy. *Ann.Surg.* **136**, 239–242.
4. SINGER, D. B. (1973) Postsplenectomy sepsis. Perspect. *Pediat.Pathol.* **1**, 285–311.
5. QUERESHI, M. A. and HAFNER, C. D. (1965) Clinical manifestations of splenic cysts: study of 75 cases. *Am.Surg.* **31**, 605–608.
6. MORGENSTERN, L. and SHAPIRO, S. J. (1980) Partial splenectomy for nonparasitic splenic cysts. *Am.Surg.* **139**, 278–281.
7. MILLAR, J. S. (1982) Partial excision and drainage of post-traumatic splenic cysts. *Br.J.Surg.* **69**, 477–478.
8. ROBINETTE, C. D. and FRAUMENI, J. F. JR. (1977) Splenectomy and subsequent mortality in veterans of the 1939–1945 war. *Lancet* **ii**, 127–129.
9. PEARSON, H. A. (1977) Sickle cell anaemia and severe infections due to encapsulated bacteria. *The Journal of Infectious Diseases* Suppl., 136, S25–S30.
10. SCHUMACHER, M. J. (1970) Serum immunoglobulin and transferrin levels after childhood splenectomy. *Arch.Dis.Child.* **45**, 114–117.
11. CONSTANTOPOLOUS, A., NAJJAR, V. A., WISH, J. B., NECHELES, T. H. and STOLBACH, L. L. (1973) Defective phagocytosis due to tuftsin deficiency in splenectomized subjects. *Am.J.Dis.Child* **125**, 663–665.
12. WALKER, W. (1976) Splenectomy in childhood. *Br.J.Surg.* **63**, 36–43.
13. SHERMAN, N. J. and ASCL, M. J. (1978) Conservative surgery for splenic injuries. *Paediatrics* **61**, 267–271.
14. MICHELS, M. A. (1942) The variational anatomy of the spleen and splenic artery. *Am.J.Anat.* **70**, 21.
15. MAZEL, M. S. (1945) Traumatic rupture of the spleen. *J.Pediatr.* **26**, 82–88.
16. MORGENSTERN, L. (1977) The avoidable complications of splenectomy. *Surg.Gyn.Obst.* **145**, 525–528.
17. MISHALANY, H. G., SLIM, M. S. and NAJJAR, N. E. (1979) Preservation of the injured spleen. *Br.J.Surg.* **66**, 671–672.
18. BURRINGTON, J. D. (1977) Surgical repair of a ruptured spleen in children. *Arch.Surg.* **112**, 417–419.

## Figures and Tables

**Figure 1 f1:**
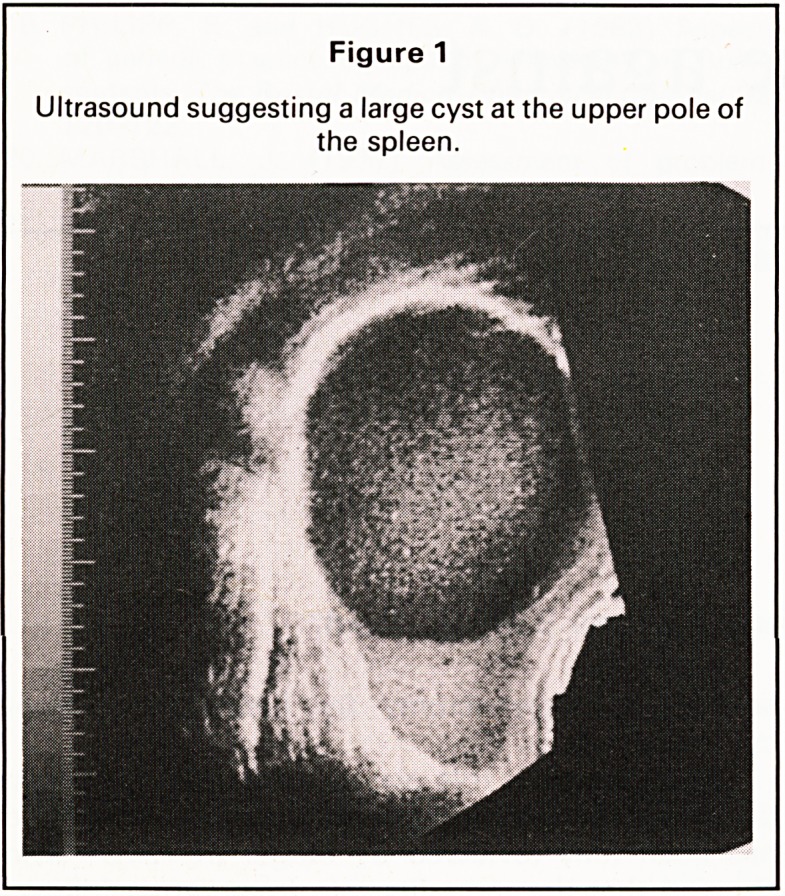


**Figure 2 f2:**
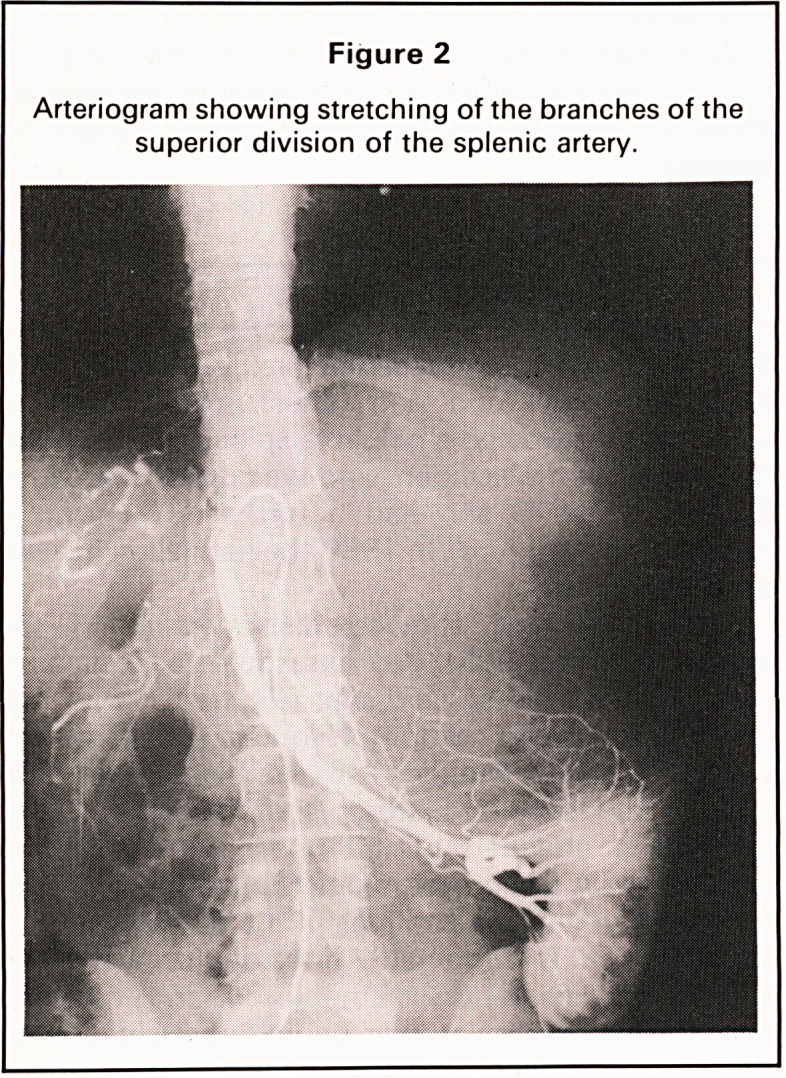


**Figure 3 f3:**
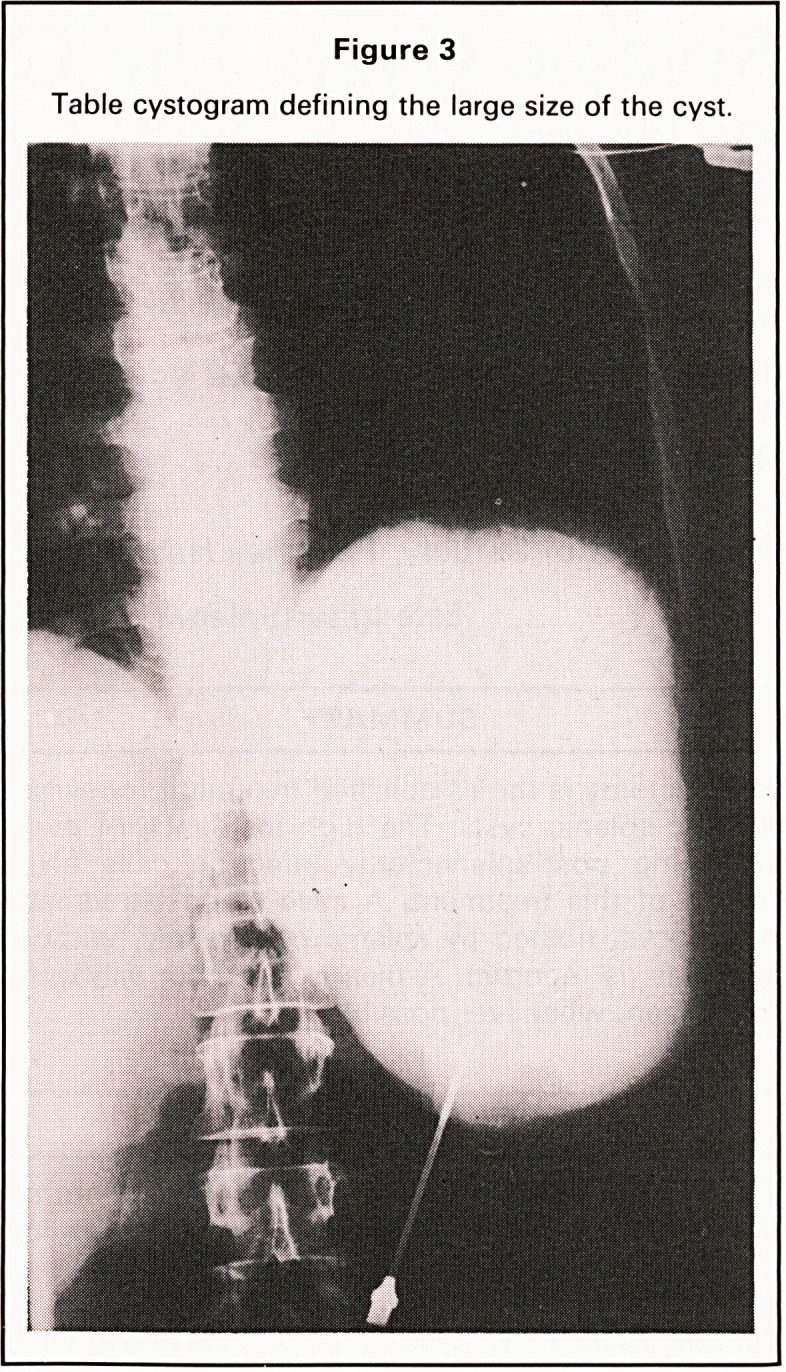


**Figure 4 f4:**
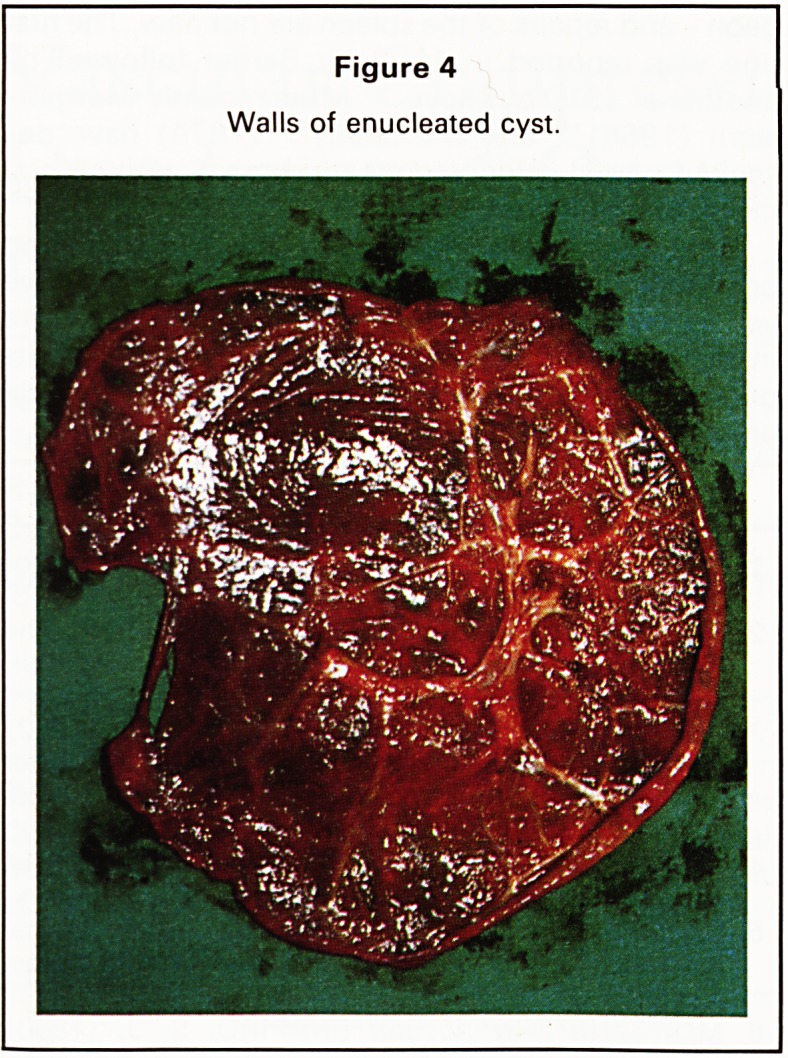


**Figure 5 f5:**
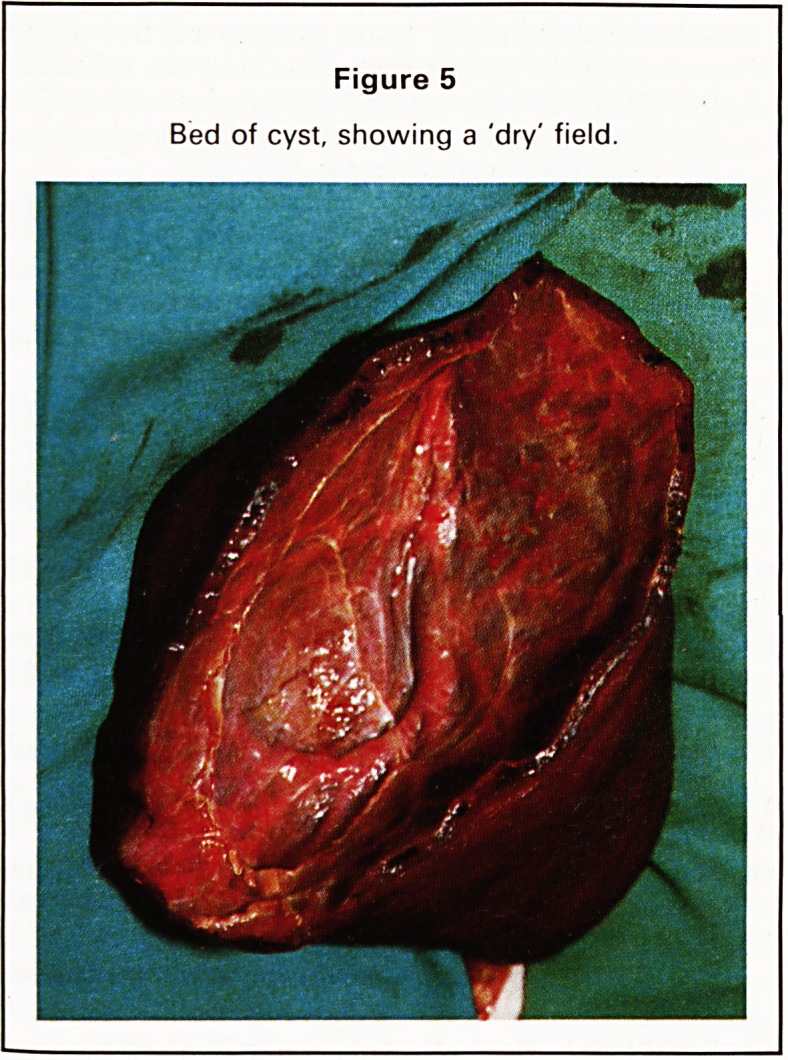


**Figure 6 f6:**